# Seasonal variations in the connection between tomato consumption and all-cause and cardio-cerebrovascular mortality

**DOI:** 10.29219/fnr.v69.12302

**Published:** 2025-09-08

**Authors:** Jiayin Lin, Jie Li, Lili Wang, Ming Cui, Liang Chen

**Affiliations:** 1 Department of Cardiology, The Second Affiliated Hospital of Dalian Medical University, Dalian, China; 2 Department of Cardiology, Ruijin Hainan Hospital Shanghai Jiao Tong University School of Medicine (Hainan Boao Research Hospital), Qionghai, China; 3 Department of Emergency, The Second Affiliated Hospital of Dalian Medical University, Dalian, China

**Keywords:** tomatoes consumption, in-season, off-season, all-cause mortality, cardio-cerebrovascular mortality

## Abstract

**Background:**

Tomatoes have full of nutritional value as well as antioxidant, anti-inflammatory, and anticancer properties. It contains substances such as lycopene and micronutrients that are beneficial to human health. Tomato consumption has been associated with reduced mortality, but the role of seasonal intake is not well understood. To address this gap, we investigated the association between in-season and off-season tomato consumption and all-cause as well as cardio-cerebrovascular mortality.

**Methods:**

This prospective study enrolled 6,260 adults from the National Health and Nutrition Examination Survey (NHANES). The endpoint events were all-cause and cardio-cerebrovascular mortality within 10 years. Cox proportional hazards analyses and competing risk modeling were employed to evaluate the influences of total and seasonal tomato consumption. Further studies were conducted on the relationship between lycopene intake and all-cause and cardio-cerebrovascular mortality over 10 years.

**Results:**

Fresh tomato consumption was significantly associated with lower all-cause mortality (hazard ratio [HR] = 0.63, 95% confidence interval [CI]: 0.45–0.87, *P* = 0.005). Moderate in-season consumption (once a week to once a day) was linked to a further reduction in all-cause mortality (HR = 0.48, 95% CI: 0.24–0.95, *P* = 0.034). Conversely, off-season consumption was associated with decreased cardio-cerebrovascular mortality (subhazard ratio [SHE] = 0.43, 95%CI: 0.23–0.79, *P* = 0.006). Moderate ketchup intake (< 1 time/day) lowered both all-cause and cardio-cerebrovascular mortality, whereas tomato juice conferred no significant survival benefit, and higher daily consumption may negate potential advantages. Elevated total and trans lycopene concentrations were also correlated with reduced mortality risks.

**Conclusion:**

Tomato consumption in different seasons shows different results with mortality: in-season intake corresponds to decreased all-cause mortality, whereas off-season intake is related to a lower risk of cardio-cerebrovascular mortality. These findings underscore the importance of considering seasonal dietary patterns in nutritional recommendations.

## Popular scientific summary

- This study revealed distinct seasonal disparities in the association between tomato consumption and mortality risk.
- Moderate intake of in-season tomatoes was associated with a reduction in all-cause mortality risk.
- Consumption of off-season tomatoes correlated with a decrease in cardio-cerebrovascular mortality risk.

Cardio-cerebrovascular disease (CVD) is one of the most common causes of death, and its incidence continues to increase steadily. It is associated with various risk factors, including diet and habits (1). Several factors increase the risk of CVD, such as inflammation, oxidative stress, and atherosclerosis. A recent meta-analysis suggested that a variety of foods (whole grains, vegetables, fruits, etc.) may mitigate the risks of coronary heart disease, heart failure, and stroke (2). Contemporary CVD prevention guidelines underscore the importance of a plant-based diet and highlight additional preventive benefits of smoking cessation and increased physical activity (3).

Tomato is an herbaceous plant of the Solanaceae family and is one of the most widely cultivated fruits and vegetables worldwide. Tomatoes are rich in lycopene, carotene, vitamins, and dietary fiber, which have a high nutritional value and are regarded as healthy foods. Tomatoes can also be processed into tomato sauce and juices. Prior evidence indicates that tomatoes exert antioxidant, anti-inflammatory, anti-atherosclerotic, and CVD-protective effects (4). Several studies showed that the consumption of tomatoes is negatively correlated with total and CVD mortality (5). Although there are significant seasonal variations in CVD incidence, especially in the winter months when the incidence is higher (6), few studies have examined the different influences of tomato consumption on CVD mortality in different seasons.

This study aimed to evaluate the impact of seasonal tomato consumption on all-cause and CVD mortality, using data from the National Health and Nutrition Examination Survey (NHANES). Through this analysis, we expect to provide targeted dietary recommendations for CVD prevention.

## Materials and methods

### Study design and population

This study utilized data sourced from the NHANES public database, which is overseen and continually updated by the National Center for Health Statistics (NCHS) and the Centers for Disease Control and Prevention in the United States. The research protocol of the NHANES was endorsed by the Ethical Committee of the NCHS. A written-informed consent was provided by all participants, granting permission for any researcher to use their data, contingent upon acknowledging the data source in the research outcomes. The NHANES datasets can be freely accessed on the official website (https://wwwn.cdc.gov/nchs/nhanes/Default.aspx).

**Fig. 1:**
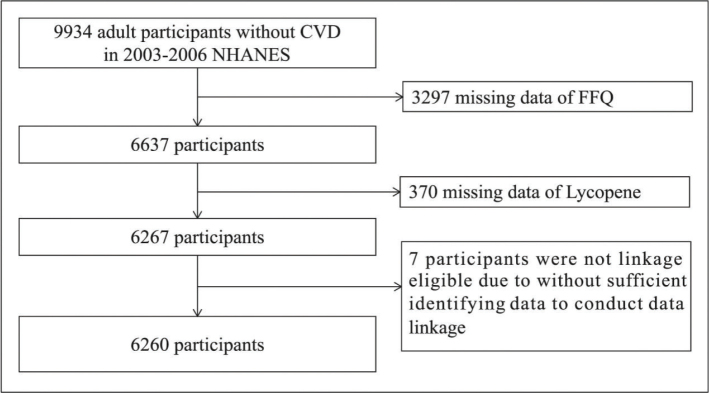
Flow chart.

From 2003 to 2006, the NHANES database included 9,934 adult participants from different races and regions, all of whom did not have CVD. CVD was defined as any history of coronary heart disease, myocardial infarction, angina pectoris, congestive heart failure, or stroke. Of these participants, 3,297 were excluded due to missing Food Frequency Questionnaire (FFQ) data, and 370 were also excluded for missing information on total and trans lycopene intake. A further 7 participants lacking sufficient follow-up data or with unknown survival status were also excluded. Ultimately, 6,260 participants were included in this study (Fig. 1).

### Food frequency questionnaire

The FFQ is a food intake questionnaire designed to collect information on the frequency of intake of various food groups in the past 12 months. In this study, the FFQ collected information of tomato intake, including in-season and off-season fresh tomatoes, as well as catsup and tomato juice frequency. In-season fresh tomatoes are defined as crops that grow naturally in the open air, usually referring to tomatoes that mature naturally from June to August. However, there will be slight differences in different regions. In the southern region, this may be advanced to May, while in the northern region, it may be delayed. The participants were divided into five groups (never, no more than once per month, once per month to once per week, once a week to once a day, and one or more times per day).

### Outcomes

The NCHS integrates survey data with death certificate records from the National Death Index (NDI) to investigate associations between various health factors and mortality outcomes. The database does not disclose mortality data for underage participants, and participant reidentification is safeguarded through data perturbation techniques applied to publicly available data. The follow-up time for deceased patients was calculated based on the quarter and year since the interview. For surviving patients, the calculation of the follow-up time was anchored to the end of the follow-up period.

### Baseline data collection

Baseline demographic and clinical information were obtained via a computer-assisted personal interview system. Variables collected included age, sex, race, education level, family income-to-poverty ratio, smoking history, alcohol use history, hypertension, hypercholesterolemia, and diabetes. Race was categorized as Mexican American, other Hispanic, non-Hispanic White, non-Hispanic Black, or other. Individuals were classified as smokers if they had smoked ≥100 cigarettes in their lifetime and as drinkers if they had consumed alcohol ≥12 times in the past 12 months. Hypertension, hypercholesterolemia, and diabetes were documented based on prior physician diagnoses.

### Statistical methods

Continuous variables are expressed as medians (first quartile and third quartile). Categorical variables are expressed as the number of cases (percentages). The effects of tomato and tomato product consumption on all-cause and cardio-cerebrovascular mortality over 10 years were analyzed using the Cox proportional hazards model. Before conducting the Cox regression, we screened all covariates for covariance, and the results suggested that the variance inflation factor of all covariates did not exceed 5. According to the principles of covariate screening, we included adjustments: comparing before and after the inclusion of the covariates, the *P*-values changed by more than 10%. The final covariates included age, sex, race, education level, ratio of family income to poverty, history of smoking, hypertension, hyperlipidemia, and diabetes. To account for the potential competing impact of other causes of death on CVD mortality, a competing risk model was performed for sensitivity analyses. All statistical analyses were conducted using SPSS software (version 27) and the R programming language (version 3.4.3, http://www.r-project.org), and *P* < 0.05, indicating that the results were statistically significant.

## Results

### Basic characteristics

A total of 6,260 adult participants without CVD were enrolled in this study with 10 years of follow-up after inclusion. Among these individuals, 5,656 (90.4%) survived, whereas 604 (9.6%) died during the observation period; 5,045 participants (80.6%) reported consuming tomatoes. The average age at death was 75.0 years, significantly higher than that of the surviving population (41.0 years). Compared with survivors, the deceased population had lower education and income levels, a higher prevalence of smoking, hypertension, hypercholesterolemia, and diabetes. Mortality was significantly associated with tomato consumption, catsup consumption, and tomato juice consumption. Both total and trans lycopene levels differed significantly between survivors and the deceased. The baseline characteristics are provided in Table 1.

**Table 1:** The baseline characteristics

Characteristic	Total	Survival	Death	*P*
Number	6,260	5,656	604	
Age, years	44.0 (29.0–62.0)	41.0 (27.0–57.0)	75.0 (64.0–80.0)	< 0.001
Sex				< 0.001
Male	2,803 (44.8)	2,461 (43.5)	342 (56.6)	
Female	3,457 (55.2)	3,195 (56.5)	262 (43.4)	
Race				< 0.001
Mexican American	1,294 (20.7)	1,212 (21.4)	82 (13.6)	
Other Hispanic	197 (3.1)	188 (3.3)	9 (1.5)	
Non-Hispanic White	3,243 (51.8)	2,848 (50.4)	395 (65.4)	
Non-Hispanic Black	1,279 (20.4)	1,171 (20.7)	108 (17.9)	
Other race	247 (3.9)	237 (4.2)	10 (1.7)	
Education level				< 0.001
Lower than high school	1,667 (26.6)	1,435 (25.4)	232 (38.4)	
High school graduate or general equivalency diploma	1,602 (25.6)	1,439 (25.4)	163 (27.0)	
Higher than high school	2,989 (47.7)	2,781 (49.2)	208 (34.4)	
Unknown	2 (0.1)	1 (0.1)	1 (0.2)	
Ratio of family income to poverty				< 0.001
Less than 1	1,133 (18.1)	1,036 (18.3)	97 (16.1)	
Between 1 and 3	2,386 (38.1)	2,077 (36.7)	309 (51.2)	
More than 3	2,458 (39.3)	2,294 (40.6)	164 (27.2)	
Unknown	283 (4.5)	249 (4.4)	34 (5.6)	
Alcohol use				0.090
No	2,481 (39.6)	2,261 (40.0)	220 (36.4)	
Yes	3,779 (60.4)	3,395 (60.0)	384 (63.6)	
Smoking				< 0.001
No	3,589 (57.3)	3,357 (59.4)	232 (38.4)	
Yes	2,671 (42.7)	2,299 (40.6)	372 (61.6)	
Hypertension				< 0.001
No	4,520 (72.2)	4,234 (74.9)	286 (47.4)	
Yes	1,740 (27.8)	1,422 (25.1)	318 (52.6)	
Hypercholesterolemia				< 0.001
No	4,658 (74.4)	4,271 (75.5)	387 (64.1)	
Yes	1,602 (25.6)	1,385 (24.5)	217 (35.9)	
Diabetes				< 0.001
No	5,801 (92.7)	5,300 (93.7)	501 (82.9)	
Yes	459 (7.3)	356 (6.3)	103 (17.1)	
Fresh tomatoes eaten				<0.001
No	810 (12.9)	770 (13.6)	40 (6.6)	
Yes	5,045 (80.6)	4,553 (80.5)	492 (81.5)	
Unknown	405 (6.5)	333 (5.9)	72 (11.9)	
Fresh tomatoes eaten in season				0.116
Never	64 (1.0)	55 (1.0)	9 (1.5)	
No more than once per month	1,081 (17.3)	972 (17.2)	109 (18.0)	
Once per month to once per week	635 (10.1)	574 (10.1)	61 (10.1)	
Once a week to once a day	2,953 (47.2)	2,672 (47.2)	281 (46.5)	
One or more times per day	634 (10.1)	553 (9.8)	81 (13.4)	
Unknown	893 (14.3)	830 (14.7)	63 (10.4)	
Fresh tomatoes eaten other times				0.107
Never	153 (2.4)	133 (2.4)	20 (3.3)	
No more than once per month	1,378 (22.0)	1,219 (21.6)	159 (26.3)	
Once per month to once per week	901 (14.4)	808 (14.3)	93 (15.4)	
Once a week to once a day	2,616 (41.8)	2,366 (41.8)	250 (41.4)	
One or more times per day	338 (5.4)	312 (5.5)	26 (4.3)	
Unknown	874 (14.0)	818 (14.5)	56 (9.3)	
Catsup				< 0.001
Never	818 (13.1)	700 (12.4)	118 (19.5)	
No more than once per month	1,554 (24.8)	1,378 (24.4)	176 (29.1)	
Once per month to once per week	986 (15.8)	894 (15.8)	92 (15.2)	
Once a week to once a day	2,556 (40.8)	2,386 (42.2)	170 (28.1)	
One or more times per day	199 (3.2)	185 (3.3)	14 (2.3)	
Unknown	147 (2.3)	113 (2.0)	34 (5.6)	
Tomato juice				< 0.001
Never	2,896 (46.3)	2,671 (47.2)	225 (37.3)	
No more than once per month	1,838 (29.4)	1,669 (29.5)	169 (28.0)	
Once per month to once per week	757 (12.1)	661 (11.7)	96 (15.9)	
Once a week to once a day	514 (8.2)	449 (7.9)	65 (10.8)	
One or more times per day	163 (2.6)	133 (2.4)	30 (5.0)	
Unknown	92 (1.5)	73 (1.3)	19 (3.1)	
Total lycopene, μg/dL	40.1 (28.0–54.4)	41.2 (29.1–55.4)	28.4 (17.3–41.4)	< 0.001
Trans lycopene, μg/dL	21.2 (14.6–29.0)	22.0 (15.3–29.6)	14.7 (8.6–21.5)	< 0.001

* Values are presented as medians or percentages. Mortality was defined as survival within 10 years of enrolment.

### Relationship between the consumption of fresh tomatoes and mortality

**Fig. 2:**
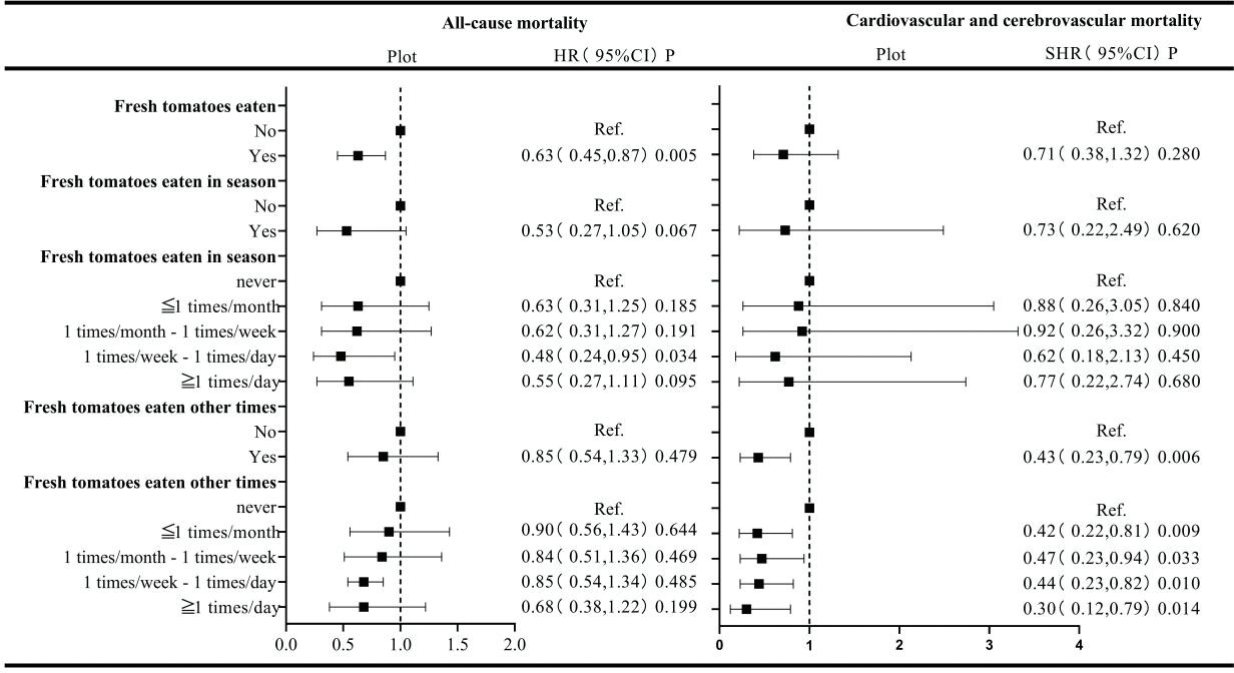
Relationship between fresh tomato consumption and mortality risk. *All-cause mortality using multivariate Cox proportional hazards analysis and cardio-cerebrovascular mortality using competing risk models. All models were adjusted for age, sex, race, education level, ratio of family income to poverty, smoking status, hypertension status, hyperlipidemia status, and diabetes status. CI, confidence interval; HR, hazard ratio; SHR, subhazard ratio; Ref., reference.

A multivariate Cox proportional hazards analysis based on the frequency of fresh tomato consumption was utilized to evaluate the relationship between fresh tomatoes and all-cause mortality. In order to exclude the competing effects of other causes of death on CVD mortality, a competing risk model was adopted. The resulted showed a significant correlation between the consumption of fresh tomatoes and all-cause mortality (hazard ratio [HR] = 0.63, 95% confidence interval [CI]: 0.45–0.87, *P* = 0.005), but no significant correlation between the consumption of fresh tomatoes and CVD mortality (Fig. 2).

### Relationship between the consumption of in-season tomatoes and mortality

The consumption of in-season tomatoes was not significantly associated with all-cause or CVD mortality. However, when evaluating specific frequency categories, participants consuming in-season fresh tomatoes ‘once a week to once a day’ experienced a notable reduction in all-cause mortality (HR = 0.48, 95% CI: 0.24–0.95, *P* = 0.034). This protective effect did not manifest in other consumption-frequency categories (Fig. 2).

### Relationship between the consumption of off-season tomatoes and mortality

The consumption of off-season tomatoes was not significantly correlated with all-cause mortality but was significantly correlated with a reduction in CVD mortality (subhazard ratio [SHR] = 0.43, 95% CI: 0.23–0.79; *P* = 0.006). Further subgroup analysis by consumption frequency revealed reduced CVD mortality in every off-season fresh tomato consumption category – specifically, ‘less than once per month’ (SHR = 0.42, 95% CI: 0.22–0.81, *P* = 0.009), ‘once per month to once per week’ (SHR = 0.47, 95% CI: 0.23–0.94, *P* = 0.033), ‘once a week to once a day’ (SHR = 0.44, 95% CI: 0.23–0.82, *P* = 0.010), and ‘more than once a day’ (SHR = 0.30, 95% CI: 0.12–0.79, *P* = 0.014) (Fig. 2).

### Relationship between the consumption of tomato products and mortality

**Fig. 3:**
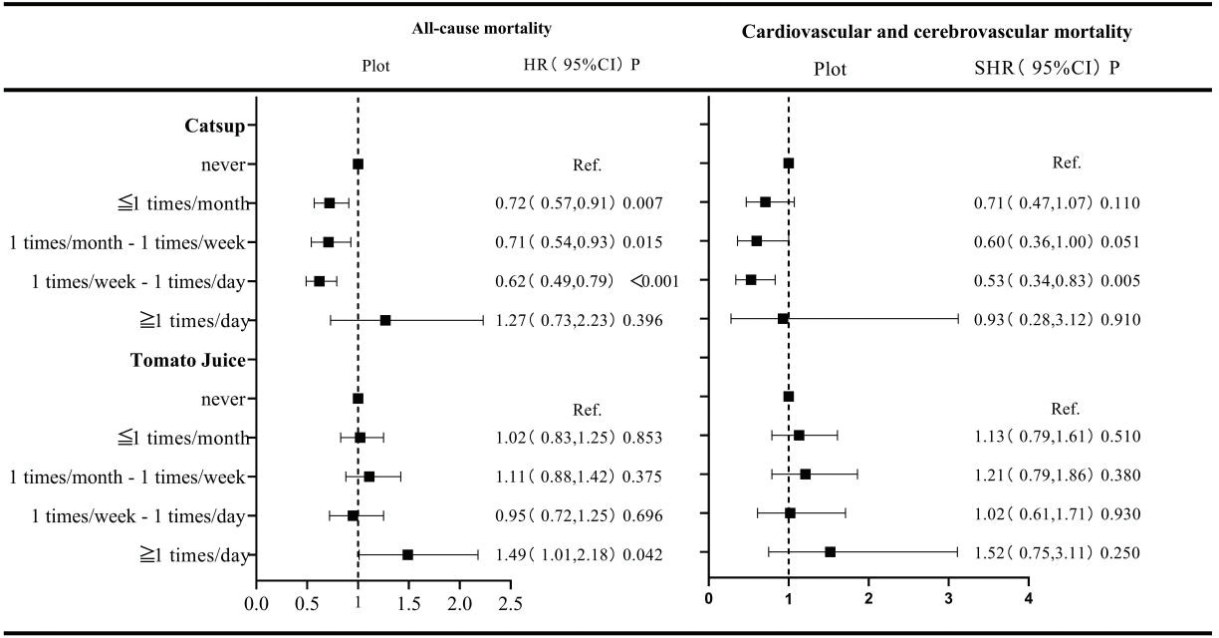
Relationship between tomato product consumption and mortality risk. *All-cause mortality using multivariate Cox proportional hazards analysis and cardio-cerebrovascular mortality using competing risk models. All models were adjusted for age, sex, race, education level, ratio of family income to poverty, smoking status, hypertension status, hyperlipidemia status, and diabetes status. CI, confidence interval; HR, hazard ratio; SHR, subhazard ratio; Ref., reference.

Moderate consumption of catsup can reduce both all-cause and CVD mortality. Specifically, catsup consumption of less than once a month (HR = 0.72, 95% CI: 0.57–0.91, *P* = 0.007), once a month to once a week (HR = 0.71, 95% CI: 0.54–0.93, *P* = 0.015), and once a week to once a day (HR = 0.62, 95% CI: 0.49–0.79, *P* < 0.001) was correlated with a low risk of all-cause mortality. Moreover, the consumption of catsup once a week to once a day reduces CVD mortality (SHR = 0.53, 95% CI: 0.34–0.83, *P* = 0.005). Tomato juice consumption more than once a day was associated with increased all-cause mortality (HR = 1.49, 95% CI: 1.01–2.18; *P* = 0.042). In comparison, there was no significant relationship between tomato juice consumption and CVD mortality (Fig. 3).

### Relationship between the lycopene intake and mortality

In light of lycopene’s key role among tomato’s nutrients, both total and trans lycopene were examined (Table 2). Total lycopene (SHR = 0.99, 95% CI: 0.98–0.99, *P* < 0.001) and trans lycopene (HR = 0.98, 95% CI: 0.97–0.99, *P* < 0.001) were each significantly associated with lower all-cause mortality. Likewise, total lycopene (SHR = 0.99, 95% CI: 0.98–1.00, *P* = 0.015) and trans lycopene (SHR = 0.98, 95% CI: 0.96–0.99, *P* = 0.008) were significantly linked to a reduced CVD mortality.

**Table 2:** Relationship between lycopene intake and mortality risk

Lycopene	All-cause mortality	Cardio-cerebrovascular mortality
	HR (95%CI) *P*	SHR (95% CI) *P*
Total lycopene	0.99 (0.98, 0.99) < 0.001	0.99 (0.98, 1.00) 0.015
Trans lycopene	0.98 (0.97, 0.99) < 0.001	0.98 (0.96, 0.99) 0.008

* All-cause mortality using multivariate Cox proportional hazards analysis and cardio-cerebrovascular mortality using competing risk models.

All models were adjusted for age, sex, race, education level, ratio of family income to poverty, smoking status, hypertension status, hyperlipidemia status, and diabetes status. CI, confidence interval; HR, hazard ratio; SHR, subhazard ratio.

## Discussion

In this study, we examined the association between fresh tomato consumption in different seasons and all-cause and CVD disease mortality based on the NHANES data. The results showed that the consumption of fresh tomatoes correlated with a reduction in all-cause mortality. Specifically, moderate in-season fresh tomato intake appeared to reduce all-cause mortality, whereas off-season fresh tomatoes were linked to decreased CVD mortality. Regarding tomato products, moderate catsup consumption was associated with reduced risks of both all-cause and CVD mortality, while tomato juice consumption exceeding one serving per day seemed to negate any survival benefit and potentially had adverse effects. In addition, this study found a clear correlation between serum lycopene concentration and mortality.

Several previous studies have shown that higher tomato consumption can positively impact various crucial health indicators, including the risk of all-cause mortality, CVD mortality, and cancer (7). The result of a study with a large sample size showed that moderate consumption of tomatoes, catsup, and lycopene correlated with a reduction in all-cause and CVD-related mortality (8). These studies support our findings and expand the understanding of the health benefits of tomatoes.

The protective mechanisms may include attenuating oxidative stress, improving lipoprotein cholesterol profiles, suppressing inflammation, and mitigating advanced glycation end product formation (8). The alpha-carotene and beta-carotene in tomatoes can reduce CVD risk factors, such as body mass index, serum triglycerides, and markers of inflammation (9). Moreover, tomato consumption can decrease the level of total cholesterol, triglycerides, and low-density lipoprotein (10). A study has also shown that consuming 300 g of tomatoes daily for 1 month can increase the level of high-density lipoprotein by 15.2% (11). In addition, the reduction in mortality caused by tomato consumption may be related to lycopene, one of the main components in tomatoes and tomato products. The average level of lycopene in tomatoes is 71.09 mg/kg, and tomatoes supply approximately 85% of dietary lycopene (1). Ross et al. indicated that lycopene has a bioavailability of approximately 92%, which is subsequently metabolized into various cis-lycopene isomers that are more readily absorbed (12). Serum lycopene concentration is closely related to tomato consumption (13). Moderate daily intake of tomatoes or tomato products can significantly boost serum lycopene levels (14), which has various beneficial effects, including lipid regulation, anti-inflammatory effects, and protection of vascular endothelial function while reducing the risk of disease and recurrence of several types of cancer (13, 15, 16). Epidemiological evidence suggests that high dietary or serum lycopene can lessen stroke risk by 26%, mortality by 37%, and CVD risk by 14% (17).

While most existing studies focus on overall tomato consumption, our findings emphasize the role of seasonal variations in tomato intake. A study showed that habitual (summer and winter) consumption of salads and fresh fruit has protective effects against cancer and CVD incidences, particularly in winter (18). Since tomatoes are often included in salads, this may have contributed to the results. However, that study’s shorter follow-up (7 years) and relatively small sample size limited validation of the seasonal element.

Previous studies on the consumption of off-season fruits and vegetables have focused mainly on differences in composition due to variations in light exposure and cultivation facilities (19). A study found that greenhouse-grown tomatoes contained significantly higher levels of vitamin C, total soluble solids, and antioxidant capacity than field-grown tomatoes (20). While changes in temperature and light had no significant effect on fruit flavor-related sugars and acids, they greatly influenced secondary metabolites. Increased irradiance elevated the levels of ascorbic acid, lycopene, β-carotene, flavonoids, and caffeic acid derivatives. Therefore, controlling fruit temperature and light may help enhance lycopene and ascorbic acid content, improving the antioxidant and anti-inflammatory effects of tomatoes, which are more beneficial for health (21). Modern greenhouse methods allow tighter control over these factors, often increasing carotenoid concentrations in off-season fresh tomatoes. For instance, tomatoes grown under LED lights have demonstrated an 18% rise in lycopene and a 142% uptick in lutein (22), while other studies have reported substantial boosts in β-carotenoids (23). Additionally, the diurnal temperature variation (DTV) may influence tomato quality and lycopene levels, with proper DTV boosting both productivity and fruit quality (24). Since DTV is uncontrollable in open-field cultivation, greenhouse-grown tomatoes may have a higher content of certain nutrients. During winter and spring, reduced availability of fresh vegetables may prompt reliance on off-season production, which could help meet nutritional demands. Thus, the positive effect of off-season fresh tomatoes on CVD health observed here may reflect both compositional enhancements and seasonal dietary gaps.

Some research suggests that CVD rates rise during winter (25), and that there is a positive correlation between cold weather and the mortality of CVD. Cold-weather factors can exacerbate CVD risk by activating the sympathetic nervous system (SNS), the renin-angiotensin system (RAS), and by increasing dehydration (26). People tend to consume richer, saltier, and higher-fat foods during winter, a pattern that encourages dyslipidemia and hypertension (27). In this context, the consumption of fruits has multiple benefits to the human body during winter. Eating fresh tomatoes in winter can provide crucial hydration, antioxidants, and vitamins (14), all of which may explain why off-season fresh tomato consumption was related to lower CVD mortality in our study. Meanwhile, lower incidence of CVD in summer and plentiful seasonal produce of fruit may obscure the effect of in-season fresh tomatoes on CVD mortality.

Hazewindus et al. suggested that catsup possesses antioxidant properties that can alleviate inflammatory responses, thereby preventing the onset of CVD (28). Similarly, our study indicates that catsup consumption was significantly correlated with lower all-cause and CVD mortality. Cooking and processing can increase cis-lycopene content of catsup, which exhibits higher bioavailability (29). This may be related to the significant association between catsup consumption and mortality observed in this study. Conversely, consuming tomato juice more than once a day appeared detrimental, possibly due to added sodium, sugar, or other additives that counteract its benefits (30). However, as limited research has addressed ketchup and tomato juice separately, future larger-scale and more detailed investigations are warranted.

Clinical trials investigating lycopene supplementation further support these observations. One randomized controlled trial (RCT) found that in cardiovascular patients, the intake of 7 mg of lycopene daily for 2 months improved brachial artery vasodilation by 53% and reduced arterial stiffness, whereas healthy participants experienced no marked changes (31). A meta-analysis likewise concluded that tomato products (70–400 g/day) or lycopene (4–30 mg/day) can significantly lower cardiovascular risk (32). Various epidemiological research works suggest that a daily intake of 2–20 mg lycopene is beneficial for preventing CVD and cancer (33). According to the dietary guidelines given by the American Heart Association and the World Health Organization, we advocate that at least 400 g of total fruits and vegetables should be consumed per day, and individuals with CVD or those at high risk may consider moderately increasing their daily consumption of tomatoes. For processed tomato products (e.g. catsup and tomato juice), it is advisable to choose low-sodium options without added sugars, which enhance lycopene bioavailability while minimizing harmful additives.

By exploring the relationships between the consumption of fresh tomatoes and mortality in different seasons, our study provides new insights about the effect of seasonal fresh tomatoes consumption on all-cause mortality and CVD mortality. Previous research has seldom examined the influence of seasonality on tomato-related health benefits. Through a comprehensive analysis of the NHANES database, the present study revealed that moderate consumption of seasonal tomatoes was significantly associated with reduced all-cause mortality and CVD mortality, a novel finding that provides new perspectives and research directions in the fields of nutrition and public health.

Nevertheless, some limitations should be noted. First, this study analyzed only American participants, which limits its generalizability and representativeness to other populations. Second, this study administered the FFQ only at baseline and was unable to control for changes in participants’ eating habits during the follow-up period. Third, the use of a single FFQ and the lack of detailed quantitative measures for tomato intake may introduce recall bias. Moreover, although we have made efforts to consider all potential confounding variables and mitigate the impact of covariates, we cannot completely rule out the factors that may affect the results. Finally, our observational design does not establish causality; experimental research will be necessary to clarify the biological mechanisms through which tomatoes and tomato products exert protective effects.

## Conclusion

Our findings highlight the impact of seasonality on the association between fresh tomato consumption and mortality. Specifically, moderate in-season fresh tomato intake correlated with reduced all-cause mortality, whereas off-season fresh tomato consumption was associated with lower CVD mortality. However, it is crucial to acknowledge that our study was observational only, and further experimental research is warranted to elucidate the underlying biological mechanisms.

## Data Availability

Researchers can freely download figures from the official NHANES website (https://www.cdc.gov/nchs/nhanes/index.htm).
